# Evaluation of large language models in a pulmonology outpatient clinic using structured clinical data and chest radiographs: a single-center prospective observational study

**DOI:** 10.3389/fmed.2026.1821639

**Published:** 2026-05-05

**Authors:** Hayriye Bektaş Aksoy, Selda Günaydın, Şaban Melih Şimşek, Ruhsel Cörüt

**Affiliations:** Department of Pulmonology, Faculty of Medicine, Giresun University, Giresun, Türkiye

**Keywords:** clinical decision support, diagnostic concordance, multimodal large language models, physician-referenced benchmarking, pulmonology

## Abstract

**Introduction:**

Large language models (LLMs) may support clinical reasoning, yet real-world outpatient studies integrating structured clinical data with chest radiographs (CXRs) remain limited. We compared three LLMs for pulmonary differential diagnosis in routine clinical practice.

**Methods:**

In this prospective, single-center observational study, consecutive adult outpatients presenting with respiratory complaints between 06 October and 31 December 2025 were enrolled. For each case, a standardized structured clinical form and de-identified CXRs were provided to three LLMs (ChatGPT-5.2, Google Gemini 3 Flash, and Microsoft Copilot) and to three blinded pulmonologists. The primary diagnosis was assigned by the examining pulmonologist, and the reference diagnosis was defined by agreement of at least two blinded pulmonologists. Concordance and Cohen’s kappa were assessed.

**Results:**

A total of 120 patients were included. Agreement among the blinded pulmonologists was high, and agreement between the primary and reference diagnoses was excellent. Compared with the reference diagnosis, ChatGPT-5.2 and Microsoft Copilot showed higher concordance than Google Gemini 3 Flash, with both demonstrating moderate overall agreement. Concordance did not differ by age or sex. Across diagnostic categories, performance was highest for pneumonia/upper respiratory tract infection and asthma.

**Discussion:**

In this real-world pulmonology outpatient cohort, ChatGPT-5.2 and Microsoft Copilot showed better diagnostic concordance than Google Gemini 3 Flash when structured clinical data and CXRs were evaluated together. These findings support the potential role of LLMs as adjunctive decision-support tools in pulmonology, while also indicating that performance remains diagnosis-dependent and insufficient to replace expert clinical judgment.

## 1 Introduction

Respiratory diseases remain among the leading causes of global morbidity and mortality, and timely, accurate diagnosis is crucial for patient outcomes. In routine pulmonology practice, diagnostic reasoning relies on the integrated interpretation of patient history, physical examination, laboratory findings, and imaging. However, the frequent non-specificity of respiratory symptoms, the presence of comorbidities that may mask or mimic pulmonary conditions, and the limitations of interpreting radiographic findings without adequate clinical context can complicate differential diagnosis.

In recent years, artificial intelligence (particularly deep learning) has achieved substantial performance improvements in thoracic imaging. Convolutional neural network models have demonstrated high accuracy in detecting clinically important pathologies on chest radiographs, including pneumonia, tuberculosis, and lung cancer (1–5). Yet much of this literature focuses on single-modality image classification, whereas real-world diagnosis is not merely an image-labeling task but a multidimensional clinical reasoning process.

Large language models (LLMs) introduce a complementary paradigm by enabling advanced understanding, synthesis, and interpretation of clinical text. Unlike image-focused artificial intelligence systems, LLMs are designed to process and generate language-based information, which makes them particularly relevant to clinical tasks that depend on textual reasoning and contextual interpretation. Evaluation studies indicate that LLMs can perform competitively in medical knowledge and question-answering tasks (6–8). Additional benchmarking studies have also reported promising performance of LLMs in medical question-answering settings across different evaluation frameworks and languages (9, 10). Specialty-specific evaluations further suggest that LLMs may approach expert-level clinical knowledge in selected domains, such as ophthalmology (11). However, performance in realistic clinical scenarios may vary, with concerns regarding diagnostic accuracy, consistency, and alignment with clinician judgment across model families and prompting or case formats (8, 12–14). Moreover, comparative studies suggest meaningful performance differences between LLM architectures and vendors when evaluated on the same clinical material (15–17).

More recently, multimodal approaches combining structured clinical data and imaging have shown potential to support more holistic diagnostic modeling (18, 19). Nevertheless, real-world outpatient studies that evaluate multiple LLMs on the same patient cohort, integrate structured clinical variables with chest radiographs, and systematically benchmark model outputs against physician diagnoses using a rigorously defined reference standard remain limited. This gap is especially relevant in pulmonology, where diagnostic decisions often depend on the combined interpretation of symptoms, exposure history, physical examination findings, and chest imaging. Evaluating LLMs in such a multimodal setting may therefore provide a more clinically meaningful perspective than text-only or vignette-based assessments.

Accordingly, the present study examines the performance of different LLMs for pulmonary differential diagnosis using real-world outpatient data in which structured clinical information and chest radiograph findings are evaluated together. Defining the reference diagnosis through multi-physician assessment enables more consistent and methodologically balanced comparisons and helps reduce bias in benchmarking. In addition, the present study may help clarify the potential contribution of LLMs to real-world clinical decision support, rather than to benchmark-style performance alone.

The primary aim of this study is to evaluate and compare the diagnostic concordance of ChatGPT-5.2, Google Gemini 3 Flash, and Microsoft Copilot with both the primary clinician’s diagnosis and a reference diagnosis defined by the agreement of at least two of three blinded pulmonologists. These models were selected because they are widely accessible, multimodal-capable, and among the most visible general-purpose LLM platforms currently used in educational and clinical contexts. Comparing their performance on the same real-world outpatient material may provide practical insight into their relative strengths and limitations in pulmonology. We further analyze model-to-model and physician-to-physician concordance metrics to characterize these models’ potential as diagnostic decision-support tools in routine clinical practice.

## 2 Materials and methods

### 2.1 Study design and setting

This prospective, observational diagnostic accuracy study was conducted in the Chest Diseases Outpatient Clinic of Giresun University Training and Research Hospital, a tertiary-care center, between 06 October 2025 and 31 December 2025.

### 2.2 Sampling method

A consecutive sampling approach was used. During the study period, all adult patients presenting to the pulmonology outpatient clinic with at least one respiratory complaint were screened for eligibility and enrolled if they met the inclusion criteria and agreed to participate.

### 2.3 Inclusion criteria

Patients aged 18 years or older who presented with at least one respiratory complaint (e.g., cough, sputum, dyspnea, wheeze, or back pain) and met the study requirements were eligible for inclusion.

### 2.4 Exclusion criteria

Patients aged <18 years, pregnant individuals, patients referred from another center with a preliminary or definitive diagnosis, those presenting solely to show test results, individuals with cognitive impairment precluding reliable history-taking, and patients who declined participation were excluded.

### 2.5 Physician assessment and diagnostic definitions

Four board-certified pulmonologists participated: one primary clinician and three blinded clinicians. All physicians had at least 5 years of specialist experience and were active in clinical practice. The primary clinician performed direct clinical assessments, including detailed history-taking, physical examination, and review of laboratory tests and radiologic findings when deemed necessary. The three blinded clinicians did not examine patients in person. They assessed only the structured clinical data forms and the de-identified chest radiographs. Blinded clinicians did not know the patients, were unaware of which patients were enrolled, had no access to one another’s assessments, and were blinded to the primary clinician’s diagnosis. To reduce diagnostic bias, two diagnostic definitions were used:

- Primary diagnosis: the diagnosis assigned by the examining primary clinician.
- Reference diagnosis: the diagnosis agreed upon independently by at least two of the three blinded clinicians.

Cases without agreement between at least two blinded clinicians were classified as “non-consensus cases” and were excluded from analyses requiring a reference diagnosis.

### 2.6 Data collection

All patients were evaluated using a standardized structured data form. Predefined symptom queries beyond routine documentation were recorded systematically. The form included:

- Demographics (age, sex).
- Respiratory symptoms (duration/pattern of cough; sputum amount/color; dyspnea at rest/exertion; wheeze; hemoptysis; chest and back pain).
- Upper respiratory tract symptoms.
- Association of symptoms with allergen exposure.
- Smoking and biomass exposure.
- ACE inhibitor use.
- History of chronic lung disease and inhaler therapy.
- Comorbidities.
- Physical examination findings of the respiratory and upper airway systems.

Chest radiography, spirometry, and laboratory testing were performed when clinically indicated. Chest radiographs were initially reviewed by the primary clinician and then de-identified before being shared with the blinded clinicians.

Chest radiographs were de-identified by masking all patient- and institution-identifying information (e.g., name, institution, identifiers) and were provided to the models as images for evaluation. De-identification was limited to occluding identifying fields in order to avoid altering diagnostic image content.

### 2.7 Large language models evaluation

The same structured clinical data and de-identified chest radiographs provided to the blinded clinicians were presented in an identical format to three LLM-based systems: ChatGPT-5.2, Google Gemini 3 Flash, and Microsoft Copilot. Each model generated a single diagnosis per case, and outputs were recorded separately. Models were run independently.

### 2.8 Diagnostic categorization

Diagnoses were grouped into 11 predefined categories: lung cancer, acute bronchitis, allergic rhinitis, asthma (including exacerbations), bronchiectasis, chronic obstructive pulmonary disease (COPD) (including exacerbations), pneumonia, post-infectious cough, upper respiratory tract infection, other pulmonary diseases, and extrapulmonary diseases. “Other pulmonary diseases” included less common respiratory conditions not captured in the predefined groups, whereas “extrapulmonary diseases” included non-respiratory causes presenting with respiratory complaints.

### 2.9 Outcomes

The primary outcome was diagnostic concordance of each LLM with the reference diagnosis. Secondary outcomes included concordance between LLMs and the primary diagnosis, agreement among blinded clinicians, and concordance stratified by diagnostic categories.

### 2.10 Statistical analysis

Statistical analyses were performed using IBM SPSS Statistics (Version 27.0; IBM Corp., Armonk, NY, United States). Continuous variables are presented as mean +/− standard deviation, and categorical variables as counts and percentages. Agreement among the three blinded clinicians was assessed using Fleiss’ kappa. Agreement between LLMs and the reference diagnosis, and between LLMs and the primary diagnosis, was assessed using Cohen’s kappa with 95% confidence intervals. Agreement levels were interpreted according to the Landis and Koch classification.

Concordance rates across LLMs were compared using the chi-square test; for statistically significant findings, multiple-testing adjustment for pairwise comparisons was performed using the Bonferroni correction. Age differences between concordant and discordant cases were evaluated using the independent-samples *t*-test, and associations between sex and concordance status were assessed using the chi-square test. All tests were two-sided, and *p* < 0.05 was considered statistically significant.

No formal sample size calculation was performed. The study was designed as a prospective real-world observational study, and consecutive eligible patients presenting during the predefined study period were included.

### 2.11 Ethics

The study was conducted in accordance with the Declaration of Helsinki. Written informed consent was obtained from all participants. Data were anonymized prior to analysis, and patient confidentiality was protected. The study protocol was approved by the Scientific Research Ethics Committee of Giresun Training and Research Hospital (Decision No: 03.09.2025/08; Date: 9 September 2025).

## 3 Results

### 3.1 Study population and inter-rater agreement

A total of 120 patients were included in the final analysis. Agreement among the three blinded pulmonologists was high. Agreement between the primary clinician and the blinded clinicians was also high, and concordance between the primary diagnosis and the reference diagnosis was excellent (Table 1).

**Table 1:** Inter-rater agreement across physician and large language models diagnoses.

Comparison	Kappa (95% CI)	*p*-value
Blinded Physicians	0.880 (0.838–0.921)	<0.001
Primary vs. Blinded Physicians	0.852 (0.823–0.882)	<0.001
Primary Diagnosis vs. Reference Diagnosis	0.915 (0.878–0.941)	<0.001
ChatGPT vs. Reference Diagnosis	0.581 (0.483–0.679)	<0.001
Google Gemini vs. Reference Diagnosis	0.393 (0.295–0.491)	<0.001
Microsoft Copilot vs. Reference Diagnosis	0.554 (0.452–0.656)	<0.001
ChatGPT vs. Google Gemini	0.577 (0.479–0.675)	<0.001
ChatGPT vs. Microsoft Copilot	0.600 (0.500–0.700)	<0.001
Google Gemini vs. Microsoft Copilot	0.535 (0.437–0.633)	<0.001

Values are presented as kappa coefficients with corresponding 95% confidence intervals (95% CI). Fleiss’ kappa was used to assess multi-rater agreement among the three blinded physicians and across all physician raters. Cohen’s kappa was used for pairwise agreement analyses. Agreement strength was interpreted according to the Landis and Koch benchmark scale. All *p*-values are two-tailed.

### 3.2 Concordance between large language models and physician diagnoses

When compared with both the primary diagnosis and the reference diagnosis, ChatGPT-5.2 and Microsoft Copilot showed higher concordance than Google Gemini 3 Flash (Table 2). Agreement with the reference diagnosis was moderate for ChatGPT-5.2 and Microsoft Copilot, whereas Google Gemini 3 Flash showed lower agreement. Pairwise agreement among the three LLMs was statistically significant in all comparisons. Significant differences in concordance proportions were observed between Google Gemini 3 Flash and Microsoft Copilot for comparisons with the primary diagnosis, and between ChatGPT-5.2 and Google Gemini 3 Flash for comparisons with the reference diagnosis.

**Table 2:** Concordance rates between large language models and physician diagnoses.

Comparison group	Concordant	Discordant	*p*-value
Primary Diagnosis vs. Reference Diagnosis	109 (90.8)	11 (9.2)	—
Primary Diagnosis vs. Large Language Models			
ChatGPT	74 (61.7)	46 (38.3)	0.016^*^
Google Gemini	56 (46.7)	64 (53.3)	
Microsoft Copilot	76 (63.3)	44 (36.7)	
Reference Diagnosis vs. Large Language Models			
ChatGPT	78 (65.0)	42 (35.0)	0.015^**^
Google Gemini	58 (48.3)	62 (51.7)	
Microsoft Copilot	76 (63.3)	44 (36.7)	

^*^Differences between Google Gemini 3 Flash vs. Microsoft Copilot. ^**^Differences between ChatGPT 5.2 and Google Gemini 3 Flash. Concordance rates were compared using the chi-square test with Bonferroni-adjusted pairwise comparisons.

### 3.3 Association of age and sex with concordance status

No significant differences in age were observed between concordant and discordant cases for any of the LLMs, whether compared with the primary diagnosis or the reference diagnosis. Likewise, concordance status was not significantly associated with sex in any comparison (Table 3).

**Table 3:** Comparison of age and sex according to concordance status between large language models and physician diagnoses.

Large language model	Concordance status	Age (years)	*p*-value (age)	Female	Male	*p*-value (sex)
Primary diagnosis						
ChatGPT	Concordant	51.0 ± 17.3	0.443	45 (66.2)	29 (55.8)	0.245
	Discordant	53.4 ± 18.5		23 (33.8)	23 (44.2)	
Google Gemini	Concordant	49.6 ± 17.4	0.228	34 (50.0)	22 (42.3)	0.403
	Discordant	53.9 ± 17.9		34 (50.0)	30 (57.7)	
Microsoft Copilot	Concordant	51.8 ± 17.2	0.681	46 (67.6)	30 (57.7)	0.262
	Discordant	52.1 ± 18.8		22 (32.4)	22 (42.3)	
Reference diagnosis						
ChatGPT	Concordant	52.2 ± 16.9	0.439	48 (70.6)	30 (57.7)	0.142
	Discordant	53.3 ± 19.4		20 (29.4)	22 (42.3)	
Google Gemini	Concordant	50.0 ± 17.1	0.301	36 (52.9)	22 (42.3)	0.248
	Discordant	53.7 ± 18.3		32 (47.1)	30 (57.7)	
Microsoft Copilot	Concordant	52.1 ± 17.2	0.922	48 (70.6)	28 (53.8)	0.059
	Discordant	51.6 ± 18.9		20 (29.4)	24 (46.2)	

Values are presented as mean ± SD for age and *n* (%) for sex. Age was compared using the independent samples *t*-test and sex using the chi-square test.

### 3.4 Distribution of diagnostic categories

According to the primary classification, asthma was the most frequent diagnosis, followed by COPD, upper respiratory tract infection, and post-infectious cough. The reference classification showed a similar distribution. Overall, the diagnostic distributions of ChatGPT-5.2 and Microsoft Copilot were broadly consistent with the physician classifications, whereas Google Gemini 3 Flash assigned a relatively higher proportion of cases to the “other pulmonary diseases” category (Table 4).

**Table 4:** Distribution of diagnostic categories across primary and reference classifications and large language models.

Diagnostic category	Primary diagnosis	Reference diagnosis	ChatGPT	Gemini	Copilot
Lung cancer	4 (3.3)	3 (2.5)	1 (0.8)	0 (0)	0 (0)
Acute bronchitis	10 (8.3)	11 (9.2)	11 (9.2)	4 (3.3)	11 (9.2)
Allergic rhinitis	4 (3.3)	5 (4.2)	1 (0.8)	4 (3.3)	3 (2.5)
Asthma (including AE)	44 (36.7)	43 (35.8)	38 (31.7)	33 (27.5)	41 (34.2)
Bronchiectasis	3 (2.5)	3 (2.5)	3 (2.5)	2 (1.7)	0 (0)
COPD (including AE)	14 (11.7)	16 (13.3)	10 (8.3)	13 (10.8)	15 (12.5)
Pneumonia	4 (3.3)	4 (3.3)	11 (9.2)	9 (7.5)	6 (5.0)
Post-infectious cough	12 (10.0)	11 (9.2)	8 (6.7)	4 (3.3)	9 (7.5)
Upper respiratory tract infection	12 (10.0)	13 (10.8)	15 (12.5)	16 (13.3)	13 (10.8)
Other pulmonary diseases	4 (3.3)	3 (2.5)	11 (9.2)	22 (18.3)	11 (9.2)
Extrapulmonary diseases	9 (7.5)	8 (6.7)	11 (9.2)	13 (10.8)	11 (9.2)
Total	120 (100)	120 (100)	120 (100)	120 (100)	120 (100)

Values are presented as number (percentage). The primary classification refers to the diagnosis assigned by the examining pulmonologist. The reference classification was defined as the diagnosis assigned by at least two of the three blinded pulmonologists. COPD, chronic obstructive pulmonary disease.

### 3.5 Category-specific concordance

Category-specific analysis showed the highest concordance across LLMs for pneumonia and upper respiratory tract infection. Concordance was also relatively high for asthma, particularly for ChatGPT-5.2 and Microsoft Copilot. In contrast, lower concordance was observed in less frequent diagnostic categories, including lung cancer and other pulmonary diseases. Overall, concordance with the reference diagnosis was highest for ChatGPT-5.2, followed closely by Microsoft Copilot, and lowest for Google Gemini 3 Flash (Table 5).

**Table 5:** Category-specific concordance rates of large language models according to primary and reference diagnoses.

Diagnostic category	Classification type	*n*	ChatGPT	Google Gemini	Microsoft Copilot
Lung cancer	Primary diagnosis	4	0 (0)	1 (25)	0 (0)
	Reference diagnosis	3	0 (0)	1 (33.3)	0 (0)
Acute bronchitis	Primary diagnosis	10	4 (40.0)	1 (10.0)	5 (50.0)
	Reference diagnosis	11	5 (45.5)	2 (18.2)	6 (54.5)
Allergic rhinitis	Primary diagnosis	4	1 (25.0)	0 (0)	2 (50.0)
	Reference diagnosis	5	1 (20.0)	0 (0)	2 (40.0)
Asthma (including AE)	Primary diagnosis	44	30 (68.2)	25 (56.8)	32 (72.7)
	Reference diagnosis	43	31 (72.1)	25 (58.1)	31 (72.1)
Bronchiectasis	Primary diagnosis	3	2 (66.7)	1 (33.3)	0 (0)
	Reference diagnosis	3	2 (66.7)	1 (33.3)	0 (0)
COPD (including AE)	Primary diagnosis	14	8 (57.1)	6 (42.9)	9 (64.3)
	Reference diagnosis	16	9 (56.3)	7 (43.8)	9 (56.3)
Pneumonia	Primary diagnosis	4	4 (100)	4 (100)	3 (75.0)
	Reference diagnosis	4	4 (100)	4 (100)	3 (75.0)
Post-infectious cough	Primary diagnosis	12	7 (58.3)	3 (25.0)	7 (58.3)
	Reference diagnosis	11	7 (63.6)	3 (27.3)	7 (63.6)
Upper respiratory tract infection	Primary diagnosis	12	10 (83.3)	10 (83.3)	10 (83.3)
	Reference diagnosis	13	11 (84.6)	10 (76.9)	10 (76.9)
Other pulmonary diseases	Primary diagnosis	4	1 (25.0)	1 (25.0)	1 (25.0)
	Reference diagnosis	3	0 (0)	0 (0)	1 (33.3)
Extrapulmonary diseases	Primary diagnosis	9	7 (77.8)	4 (44.4)	7 (77.8)
	Reference diagnosis	8	8 (100)	5 (62.5)	7 (87.5)
Total	Primary diagnosis	120	74 (61.7)	56 (46.7)	76 (63.3)
	Reference diagnosis	120	78 (65.0)	58 (48.3)	76 (63.3)

Values are presented as number (percentage). For each category, percentages represent the proportion of cases in which the large language model assigned the same diagnosis as the primary classification or the reference classification. COPD, chronic obstructive pulmonary disease.

## 4 Discussion

Deep learning-based systems have shown substantial progress in thoracic imaging, and multiple studies have reported high diagnostic accuracy for chest radiograph-based detection of specific conditions (5, 7, 20–22). In particular, convolutional neural network models have been reported to achieve near-radiologist-level performance for focused tasks such as identifying pneumonia, tuberculosis, and lung cancer on chest radiographs (20, 21). However, much of this literature has evaluated single-modality image classification with a limited set of diagnostic labels. In routine clinical practice, diagnostic reasoning extends beyond radiographic pattern recognition and requires integration of clinical history, symptom duration, risk factors, and physical examination findings within a patient-specific context.

In this study, we evaluated three LLMs-ChatGPT-5.2, Google Gemini 3 Flash, and Microsoft Copilot-using real-world pulmonology outpatient data in which structured clinical information and chest radiograph findings were assessed together. Using both the primary clinician’s diagnosis and a multi-physician reference diagnosis, we quantified concordance and agreement metrics across models and clinicians. Overall, our findings show that LLMs can demonstrate measurable agreement with physician-based diagnoses; however, concordance with the reference diagnosis remained moderate and differed across models. More importantly, these findings suggest that current LLMs may offer supportive value in pulmonology practice, but they are not yet sufficiently aligned with physician-derived diagnoses to support independent clinical use.

Previous work on LLMs has often relied on examination-style questions, curated vignettes, or structured question-answer formats (8, 16). Although such studies are informative, they may not fully reflect the variability and complexity of real outpatient practice. In contrast, performance in real clinical settings may be more variable, and reductions in diagnostic accuracy and consistency have been reported in complex presentations or in cases with multiple comorbidities (4, 14, 15, 23, 24). Our results are consistent with these observations and indicate that performance remains context-dependent when LLMs are applied to routine outpatient pulmonary assessment.

An important finding of the present study is that diagnostic concordance was not uniform across models. ChatGPT-5.2 and Microsoft Copilot showed higher concordance with the reference diagnosis than Google Gemini 3 Flash. This observation is in line with reports demonstrating heterogeneity in diagnostic performance and consistency across LLM architectures and vendors when evaluated on comparable clinical material (13, 25, 26). At the same time, these differences should not be interpreted as definitive evidence of superiority, since performance may be influenced by several technical factors, including training data characteristics, update frequency, prompting sensitivity, and the structure and completeness of the clinical input. For this reason, model selection and clinical integration should be guided by use-case-specific validation rather than by a single summary metric alone.

We also found high agreement between the primary clinician and the blinded pulmonologists, supporting the internal consistency of expert clinical assessment in this real-world setting. This finding reinforces the central role of contextual clinical judgment and integrative reasoning in pulmonology practice. In turn, the comparatively lower agreement observed for the LLMs supports positioning these systems, at present, as assistive tools under human oversight rather than autonomous diagnostic decision-makers in routine workflows.

Methodologically, this study contributes to the literature by using routine outpatient data and by defining a reference diagnosis through multi-physician assessment, allowing a more structured and balanced benchmark. Whereas many previous investigations have evaluated LLMs using selected case sets or exam questions, our approach reflects real-world clinical information captured during routine care and therefore provides a more pragmatic perspective on potential clinical utility.

Moderate agreement should not be interpreted as absence of clinical relevance. Rather, the main implication of these findings is that LLMs may be useful in supportive roles, such as helping structure differential diagnosis lists, prompting consideration of alternative diagnoses, and assisting clinicians in organizing their reasoning, particularly when multimodal inputs are incorporated. Future work integrating richer multimodal data, improved sensitivity to clinical context, and domain-specific adaptation may further improve performance and reliability (27–29).

Overall, our findings suggest that LLMs hold promise as decision-support tools in pulmonology; however, at present, they do not match physician-derived reference diagnoses closely enough to replace expert evaluation. Their most appropriate role currently appears to be adjunctive support within clinician-led care, and continued validation, careful definition of intended use, and ongoing human oversight remain essential for safe clinical implementation.

## 5 Limitations

This study has several limitations. First, it was conducted as a prospective observational study in a single-center pulmonology outpatient cohort, which may limit the generalizability of the findings to other clinical settings and populations. Second, although the reference diagnosis was defined through blinded multi-physician assessment, a universal gold standard was not available for all cases; therefore, the results should be interpreted primarily as agreement with physician-derived classifications rather than as definitive diagnostic accuracy. Third, some diagnostic categories included only a small number of cases, such as lung cancer, bronchiectasis, and other infrequent conditions, which may have increased variability in category-specific estimates.

In addition, LLM outputs may be influenced by model versioning, update cycles, and prompting strategy. Because the evaluation was performed within a defined time window using a specific prompt structure, performance may differ with later model updates or alternative prompting approaches. Chest radiographs were provided to the models as anonymized images, and variability in image quality or acquisition technique, as well as potential information loss related to masking during anonymization, may also have affected model outputs. The evaluation was further limited to chest radiography and did not address scenarios in which additional imaging modalities, such as computed tomography, might have provided incremental diagnostic value.

Finally, given the observational design, we assessed diagnostic concordance but did not evaluate downstream clinical outcomes, workflow impact, or safety-related endpoints. Accordingly, no causal inference can be made regarding clinical benefit within the scope of this study.

## 6 Conclusion

In this prospective observational study, we comparatively assessed the diagnostic concordance of ChatGPT-5.2, Google Gemini 3 Flash, and Microsoft Copilot for pulmonary differential diagnosis using real-world outpatient data, benchmarked against both the primary clinician’s diagnosis and a multi-physician reference diagnosis. Agreement with the reference diagnosis was moderate, with meaningful differences across models; ChatGPT-5.2 and Microsoft Copilot showed higher concordance than Google Gemini 3 Flash. These findings indicate that LLM-based systems may offer supportive value as decision-support tools in pulmonology, but they do not currently achieve sufficient alignment with physician-derived diagnoses to replace expert evaluation. External validation, clear definition of intended use, and continued human oversight remain essential for safe clinical implementation.

## Data Availability

The original contributions presented in the study are included in the article/supplementary material, further inquiries can be directed to the corresponding author.

## References

[1] Ippolito D Maino C Gandola D Franco PN Miron R Barbu V . Artificial intelligence applied to chest X-ray: a reliable tool to assess the differential diagnosis of lung pneumonia in the emergency department. Diseases. (2023) 11:171. doi: 10.3390/diseases11040171, 37987282 PMC10660530

[2] Colquitt J Jordan M Court R Loveman E Parr J Ghosh I . Artificial intelligence software for analysing chest X-ray images to identify suspected lung cancer: an evidence synthesis early value assessment. Health Technol Assess. (2024) 28:1–75. doi: 10.3310/LKRT4721, 39254229 PMC11403378

[3] Becker J Decker JA Rommele C Kahn M Messmann H Wehler M . Artificial intelligence-based detection of pneumonia in chest radiographs. Diagnostics. (2022) 12:1465. doi: 10.3390/diagnostics12061465, 35741276 PMC9221818

[4] de Camargo TFO Ribeiro GAS da Silva MCB Silva LO Torres P Rodrigues D . Clinical validation of an artificial intelligence algorithm for classifying tuberculosis and pulmonary findings in chest radiographs. Front Artif Intell. (2025) 8:1512910. doi: 10.3389/frai.2025.1512910, 39991462 PMC11843218

[5] Lakhani P Sundaram B. Deep learning at chest radiography: automated classification of pulmonary tuberculosis by using convolutional neural networks. Radiology. (2017) 284:574–82. doi: 10.1148/radiol.2017162326, 28436741

[6] Singhal K Azizi S Tu T Mahdavi SS Wei J Chung HW . Large language models encode clinical knowledge. Nature. (2023) 620:172–80. doi: 10.1038/s41586-023-06291-2, 37438534 PMC10396962

[7] Singhal K Tu T Gottweis J Sayres R Wulczyn E Amin M . Toward expert-level medical question answering with large language models. Nat Med. (2025) 31:943–50. doi: 10.1038/s41591-024-03423-7, 39779926 PMC11922739

[8] Levine DM Tuwani R Kompa B Varma A Finlayson SG Mehrotra A . The diagnostic and triage accuracy of the GPT-3 artificial intelligence model: an observational study. Lancet Digit Health. (2024) 6:e555–61. doi: 10.1016/S2589-7500(24)00097-9, 39059888

[9] Lucas MM Yang J Pomeroy JK Yang CC. Reasoning with large language models for medical question answering. J Am Med Inform Assoc. (2024) 31:1964–75. doi: 10.1093/jamia/ocae131, 38960731 PMC11339506

[10] Alonso I Oronoz M Agerri R. MedExpQA: multilingual benchmarking of large language models for medical question answering. Artif Intell Med. (2024) 155:102938. doi: 10.1016/j.artmed.2024.102938, 39121544

[11] Thirunavukarasu AJ Mahmood S Malem A Foster WP Sanghera R Hassan R . Large language models approach expert-level clinical knowledge and reasoning in ophthalmology: a head-to-head cross-sectional study. PLoS Digit Health. (2024) 3:e0000341. doi: 10.1371/journal.pdig.0000341, 38630683 PMC11023493

[12] Hirosawa T Harada Y Mizuta K Sakamoto T Tokumasu K Shimizu T. Diagnostic performance of generative artificial intelligences for a series of complex case reports. Digit Health. (2024) 10:20552076241265215. doi: 10.1177/20552076241265215, 39229463 PMC11369864

[13] Sonoda Y Kurokawa R Nakamura Y Kanzawa J Kurokawa M Ohizumi Y . Diagnostic performances of GPT-4o, Claude 3 Opus, and Gemini 1.5 Pro in “Diagnosis Please” cases. Jpn J Radiol. (2024) 42:1231–5. doi: 10.1007/s11604-024-01619-y, 38954192 PMC11522128

[14] Aksoy I Arslan MK. Comparison of performance of artificial intelligence tools in answering emergency medicine question pool: ChatGPT 4.0, Google Gemini and Microsoft Copilot. Pak J Med Sci. (2025) 41:968–72. doi: 10.12669/pjms.41.4.11178, 40290213 PMC12022595

[15] Huang KA Choudhary HK Hardin WM Prakash N. Comparative analysis of ChatGPT-4o and Gemini advanced performance on diagnostic radiology in-training exams. Cureus. (2025) 17:e80874. doi: 10.7759/cureus.80874, 40255788 PMC12009162

[16] Arslan B Sutasir MN Altinbilek E. Performance of Microsoft Copilot in the diagnostic process of pulmonary embolism. West J Emerg Med. (2025) 26:1030–9. doi: 10.5811/westjem.24995, 40794985 PMC12342421

[17] Bayala YLT Zabsonre/Tiendrebeogo WJS Ouedraogo DD Kabore F Sougue C Yameogo AR . Performance of the large language models in African rheumatology: a diagnostic test accuracy study of ChatGPT-4, Gemini, Copilot, and Claude artificial intelligence. BMC Rheumatol. (2025) 9:54. doi: 10.1186/s41927-025-00512-z, 40380276 PMC12083132

[18] Yang L Wan Y Pan F. Enhancing chest X-ray diagnosis with a multimodal deep learning network by integrating clinical history to refine attention. J Imaging Inform Med. (2025) 38:3568–83. doi: 10.1007/s10278-025-01446-1, 39971817 PMC12701160

[19] Pan CT Kumar R Wen ZH Wang CH Chang CY Shiue YL. Improving respiratory infection diagnosis with deep learning and combinatorial fusion: a two-stage approach using chest X-ray imaging. Diagnostics. (2024) 14:500. doi: 10.3390/diagnostics14050500, 38472972 PMC10930782

[20] Kumar R Pan CT Lin YM Yow-Ling S Chung TS Janesha UGS. Enhanced multi-model deep learning for rapid and precise diagnosis of pulmonary diseases using chest X-ray imaging. Diagnostics. (2025) 15:248. doi: 10.3390/diagnostics15030248, 39941178 PMC11817112

[21] Kim S Rim B Choi S Lee A Min S Hong M. Deep learning in multi-class lung diseases' classification on chest X-ray images. Diagnostics. (2022) 12:915. doi: 10.3390/diagnostics12040915, 35453963 PMC9025806

[22] Nasser AA Akhloufi MA. Deep learning methods for chest disease detection using radiography images. SN Comput Sci. (2023) 4:388. doi: 10.1007/s42979-023-01818-w, 37200562 PMC10173935

[23] Geric C Qin ZZ Denkinger CM Kik SV Marais B Anjos A . The rise of artificial intelligence reading of chest X-rays for enhanced TB diagnosis and elimination. Int J Tuberc Lung Dis. (2023) 27:367–72. doi: 10.5588/ijtld.22.0687, 37143227 PMC10171486

[24] Hamanaka R Oda M. Can artificial intelligence replace humans for detecting lung tumors on radiographs? An examination of resected malignant lung tumors. J Pers Med. (2024) 14:164. doi: 10.3390/jpm14020164, 38392597 PMC10890665

[25] Ueda D Matsumoto T Yamamoto A Walston SL Mitsuyama Y Takita H . A deep learning-based model to estimate pulmonary function from chest X-rays: multi-institutional model development and validation study in Japan. Lancet Digit Health. (2024) 6:e580–8. doi: 10.1016/S2589-7500(24)00113-4, 38981834

[26] Salman IM Ameer OZ Khanfar MA Hsieh YH. Artificial intelligence in healthcare education: evaluating the accuracy of ChatGPT, Copilot, and Google Gemini in cardiovascular pharmacology. Front Med. (2025) 12:1495378. doi: 10.3389/fmed.2025.1495378, 40046930 PMC11879995

[27] Ko J Park S Woo HG. Optimization of vision transformer-based detection of lung diseases from chest X-ray images. BMC Med Inform Decis Mak. (2024) 24:191. doi: 10.1186/s12911-024-02591-3, 38978027 PMC11232177

[28] Goktas P Karakaya G Kalyoncu AF Damadoglu E. Artificial intelligence Chatbots in allergy and immunology practice: where have we been and where are we going? J Allergy Clin Immunol Pract. (2023) 11:2697–700. doi: 10.1016/j.jaip.2023.05.042, 37301435

[29] Abgrall G Holder AL Chelly Dagdia Z Zeitouni K Monnet X. Should AI models be explainable to clinicians? Crit Care. (2024) 28:301. doi: 10.1186/s13054-024-05005-y, 39267172 PMC11391805

